# Exome-based genome-wide association study and risk assessment using genetic risk score to prostate cancer in the Korean population

**DOI:** 10.18632/oncotarget.16540

**Published:** 2017-03-24

**Authors:** Jong Jin Oh, Soo Ji Lee, Joo-Yeon Hwang, Dokyoon Kim, Sang Eun Lee, Sung Kyu Hong, Jin-Nyoung Ho, Sungroh Yoon, Joohon Sung, Wun-Jae Kim, Seok-Soo Byun

**Affiliations:** ^1^ Department of Urology, Seoul National University College of Medicine, Seoul National University Bundang Hospital, Seongnam, Korea; ^2^ Complex Disease and Genome Epidemiology Branch, Department of Public Health, Graduate School of Public Health, Seoul National University, Seoul, Korea; ^3^ Division of Structural & Functional genomics, Center for Genome Science, Korean National Institute of Health, KCDC, Korea; ^4^ Department of Biomedical and Translational Informatics, Geisinger Health System, Danville, PA, USA; ^5^ Biomedical Research Institute, Seoul National University Bundang Hospital, Seongnam, Korea; ^6^ Department of Electrical and Computer Engineering, Seoul National University, Seoul, Korea; ^7^ Department of Urology, Chung Buk National University Hospital, Cheongju, Korea

**Keywords:** prostate, prostate cancer, exome, Korean

## Abstract

**Purpose:**

To investigate exome-wide genetic variants associated with prostate cancer (PCa) in Koreans and evaluate the discriminative ability by the genetic risk score (GRS).

**Patients and methods:**

We prospectively recruited 1,001 PCa cases from a tertiary hospital and conducted a case-control study including 2,641 healthy men (Stage I). Participants were analyzed using HumanExome BeadChip. For the external validation, additionally enrolled 514 PCa cases and 548 controls (independent cohort) were analyzed for the identified single nucleotide polymorphisms (SNPs) of Stage I (Stage II). The GRS was calculated as a non-weighted sum of the risk allele counts and investigated for accuracy of prediction of PCa.

**Results:**

the mean age was 66.3 years, and the median level of prostate specific antigen (PSA) was 9.19 ng/ml in PCa cases. In Stage I, 4 loci containing 5 variants (rs1512268 on 8p21.2; rs1016343 and rs7837688 on 8q24.21; rs7501939 on 17q12, and rs2735839 on 19q13.33) were confirmed to reach exome-wide significance (p<8.3×10^-7^). In Stage II, the mean GRS was 4.23 ± 1.44 for the controls and 4.78 ± 1.43 for the cases. As a reference to GRS 4, GRS 6, 7 and 8 showed a statistically significant risk of PCa (OR=1.85, 2.11 and 3.34, respectively).

**Conclusions:**

The five variants were validated to associate with PCa in firstly performed exome-wide study in Koreans. The addition of individualized calculated GRS effectively enhanced the accuracy of prediction. These results need to be validated in future studies.

## INTRODUCTION

Prostate cancer (PCa) is the most common cancer diagnosed in men and is second leading cancer death among men in the United States [1] and it is 5^th^ most common cancer in South Korean men. The incidence and mortality rates of PCa vary by 25-fold and 10-fold across populations [2]. The highest incidence rates are found in Western developed countries, and the highest mortality rates are found in the African-Americans, whereas the lowest incidence and mortality rates are reported in Asians [3]. The big differences in the epidemiologic profile raise an important question whether genetic heterogeneity or differences in the environments have a greater impact on PCa development [4].

To date, genome wide meta-analyses of PCa have provided numerous common susceptibility loci from European ancestry population [5–7]. A fine-mapping study from the European-based international consortium provided additional novel PCa signals [8]. However, a large-scaled meta-analysis identified two new PCa risk loci from only two East-Asian groups (Japanese and Chinese) [3]. Since there was a genetic difference according to the ethnic groups, we performed the first Korean population-based exome-wide association study for PCa.

Cancer risk prediction by genetic risk scores (GRS) have been designed as an efficient and effective approach in terms of clinical utility [9]. Recently, genetic risk assessment studies have been reported evaluating the cumulative genetic scores for PCa risk [10, 11]. Several customized products such as Oncotype Dx® (Genomic Health Inc, Redwood City, CA, USA), Prolaris® (Myriad Genetics, Salt Lake City, UT, USA) and Decipher® (GenomeDX Biosciences, Vancouver, BC, Canada), use a concept of polygenic risk score that is currently available to the real clinical situation [12, 13]. However, the vast majority of genome wide association study (GWAS)-derived loci explain only a limited fraction of the disease risk development and functional implications as a non-coding variant. Given ethnic differences and genetic heterogeneity, integrative genetic approaches have not been fully explored in non-European populations. Taken together, exome-based association studies and risk prediction analyses are needed to understand the potential etiologic and functional mechanisms of PCa risk. Finally, we evaluated exome-based association studies of PCa in a Korean population and additionally provide a risk prediction model using GRS formation.

## RESULTS

**Figure 1:**
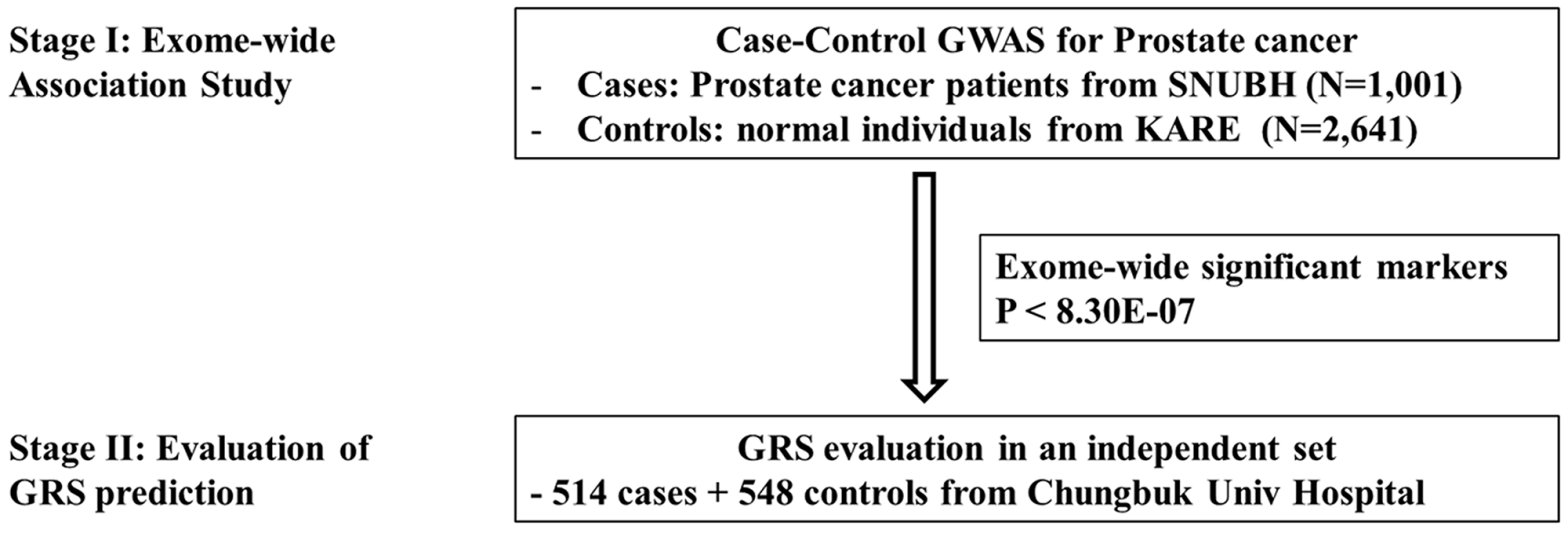
Overall study scheme

The descriptive characteristics for the discovery stage (1,001 PCa patients and 2,641 controls) and the validation stage (514 and 548) participants are shown in Table 1. All of the men were ethnical Koreans. For the cases, the mean age was 66.3 years, the median level of prostate specific antigen (PSA) was 9.19 ng/ml and the average prostate size was 37.48 cc. Most of the cases had a biopsy Gleason score of less than 8 (biopsy Gleason score 6: 39.3% and score 7: 41.1%). Of the cases, 41 were metastatic and 820 (81.9%) were treated by radical prostatectomy (RP). The controls were younger, but not substantially leaner than the cases. Our study was performed in a two-stage design as depicted in Figure 1. In Stage I, 1,001 PCa samples and 2,641 population controls were used to find the associated markers in the Korean population and examine the previously GWAS-identified SNPs in different ethnic populations. In Stage II, the GRS was calculated based on the validated SNPs from Stage I in order to determine their discriminative ability in an independent set of cases and controls.

**Table 1:** Clinical characteristics for exome-wide association study among Korean population

Variables	Prostate cancer(n = 1,001)	Control (n = 2,641)	Prostate Cancer II(n=514)	Control II (n=548)
Mean Age (yrs) ± SD	66.32 ± 6.65	50.94 ± 8.50	69.08 ± 7.56	50.04 ±13.33
Median PSA (ng/ml) ± SD	9.19 ± 138.60	N.A	10.10 ± 648.92	N.A.
Prostate volume (ml) ± SD	37.48 ± 17.35	N.A	N.A	N.A.
Body mass index (kg/m^2^) ± SD	24.47 ± 8.23	24.25 ± 3.04		
Pathologic stage (n_%)		-		
pT2	460 (56.1)	-	N.A	
pT3a	271 (33.0)	-	N.A	
pT3b	79 (9.6)	-	N.A	
pT4	10 (1.2)	-	N.A	
Gleason score (n_%)		-		
6	393 (39.3)	-	43 (8.4)	
7	411 (41.1)	-	278 (54.1)	
8	138 (13.8)	-	86 (16.7)	
9	47 (4.7)	-	95 (18.5)	
10	12 (1.2)		12 (2.3)	

Abbreviations: SD = standard deviation; PSA = prostate specific antigen

### Stage I Exome-wide association study

Among the 60,276 exonic SNPs that were available after the genotype QC, Table 2 shows the results of the exome-wide association with PCa. In this Korean population, the stage I association study detected 4 loci containing 5 lead variants that conferred exome-wide significance, commonly accepted as a p-value below 0.05 divided by the number of association tests (here, 0.05/60,276 ≈ 8.30E-07). The complete study results are presented in Supplementary Table 1 with markers of P < 10E-05.

**Table 2:** Association study results for prostate cancer that were exome-wide significant (P < 8.30 × 10-7). Results from the previous studies with genome-wide significance were presented for each SNPs

SNP	Korean Study (Stage I)							Previously reported results		
(reference/risk allele, forward)	Gene	Chromo-somal region	Risk allele frequency		OR(95%CI)	p-value	Genetic context	population	OR(95%CI)	p-value
			case	control						
rs1512268 (C/T)	NKX3-1	8p21.2	0.376	0.306	1.37 (1.23-1.53)	1.90E-08	Intergenic	UK and Australia [14]	1.18 (1.14-1.22)	2.5×10-23
								Japanese [15]	1.34	4.3×10-11
								Latinos [16]	1.21 (1.07-1.37)	2.8×10-3
rs1016343 (C/T)	PRNCR1	8q24.21	0.408	0.306	1.58 (1.42-1.77)	2.42E-16	Exonic	UK and Australia [17]	1.37	1×10-7
								European [18]	1.31 (1.20-1.42)	4×10-10
rs7837688 (G/T)	CASC8 - CASC11	8q24.21	0.213	0.136	1.73 (1.51-1.98)	2.85E-15	Intergenic	Japanese [15]	NA	1×10-25
								UK and Australia [17]	1.41	9×10-12
rs7501939* (T/C)	HNF1B	17q12	0.779	0.714	1.41 (1.25-1.61)	2.78E-08	Intronic	Japanese [15]	NA	1×10-12
								European [18]	1.19 (1.11-1.28)	2×10-6
rs2735839* (A/G)	KLK3	19q13.33	0.677	0.605	1.31 (1.19-1.47)	6.35E-07	Upstream	UK and Australia [17]	0.83 (0.75–0.91)	1.5×10-18
								European [18]	1.20 (1.10-1.33)	2×10-18
								Multi-ethnic [19]	1.20 (1.13-1.28)	2×10-18

* risk allele is the major allele

**Figure 2:**
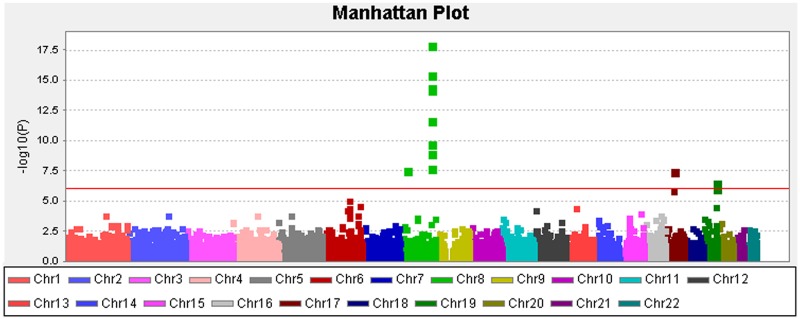
Manhattan plot depicting the Stage I results of the exome-wide association study Y-axis: -log10(p-value); X-axis: genomic position. The solid red line shows the study-specific exome-wide significance level at a p-value of 8.30E-07.

At the Stage II analysis of the construction of the GRS, two SNPs (rs1456315 and rs266849) of these 5 SNPs were replaced with adjacent SNPs (rs1016343 and rs2735839) with high LD (r > 0.9) and meaningful signal (p<10E-05), because analysis of the two SNPs failed in the Stage II samples. Three SNPs were located in 8q21-24, one in 17q12 and one in 19q13.33. All five of the hits reside at or nearby the genes that were previously identified by GWA studies; e.g., NK3-1, PRNCR1, CASC8/11, HNF1B, and KLK3 (presented in Table 2) [14–19]. The QQ plot (Supplementary Figure 1) suggested that inflation by type I errors from any cause were unlikely (inflation factor, λ=0.92 by genomic control analysis). The Manhattan plot for our analysis for PCa susceptibility loci testing the additive effects of each SNP are presented in Figure 2. Supplementary Figure 2 presents a regional plot with the LD structure for the chromosomal region 8q24.21. As indicated in the figure, the two SNPs in the 8q24.21 region were in different LD blocks.

### GRS for PCa risk prediction

**Figure 3:**
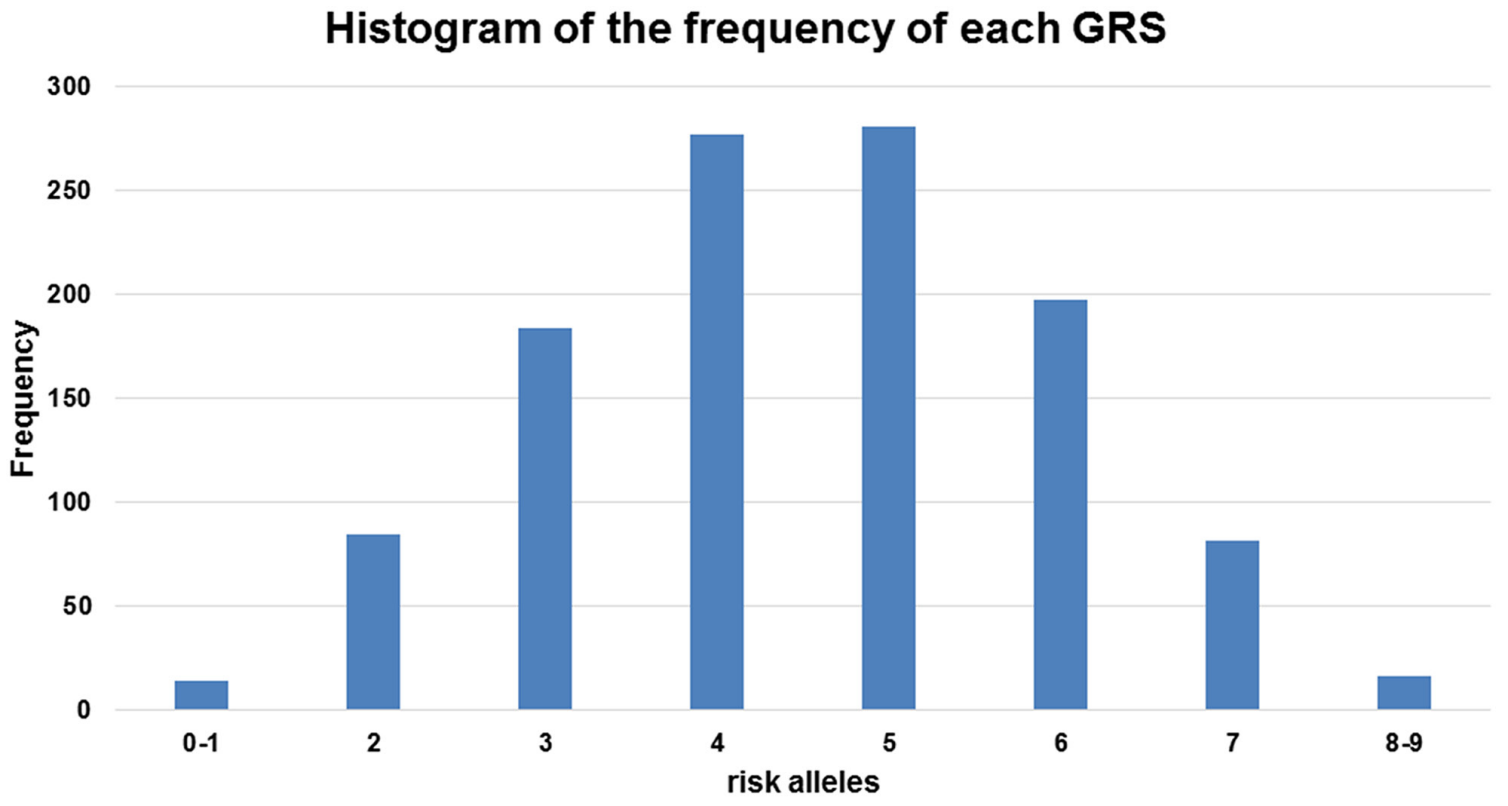
Frequency distribution across the genetic risk score (GRS) groups The histogram is plotted on the x-axis representing each GRS category as the sum of the risk alleles across the five loci, and the y-axis plots the number of individuals in each GRS category.

**Figure 4:**
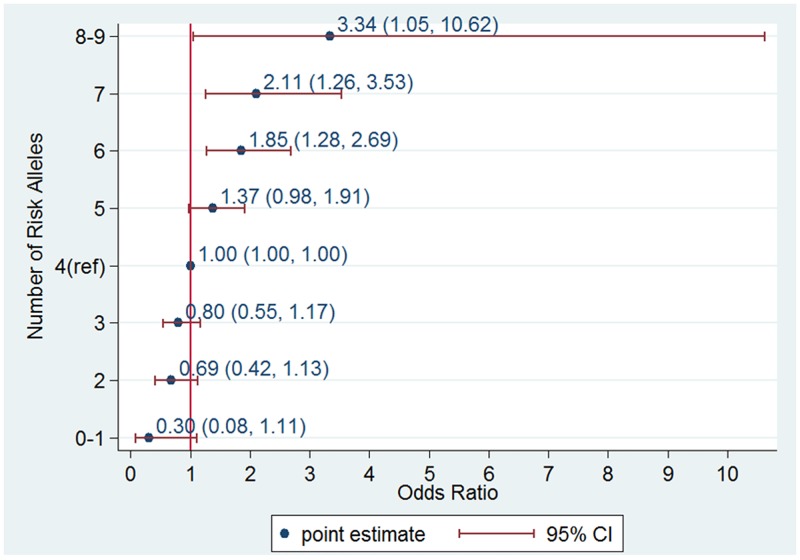
Odds ratio (OR) of prostate cancer according to genetic risk score (GRS) category in an independent data set consisting of 514 cases and 548 controls from the Chungbuk University Hospital An allele dosage category of 4 was used as a reference group.

When we used the sum of the number of risk alleles as the GRS, the distribution of the GRS followed a normal distribution (Figure 3) with a mean score of 4.23 ± 1.44 for the controls and 4.78 ± 1.43 for the cases. The GRS was associated with an increased PCa risk for the Stage II samples from the Chungbuk University Hospital (cases n=514 and controls n=548); taking the group with a GRS=4 as a reference, the higher GRS group showed an increase and the lower GRS group showed a decrease in PCa risk. A GRS score above 6 showed a statistically significant risk of PCa; OR=1.85, 95%CI [1.28, 2.69], OR 2.11, 95%CI [1.26, 3.53] and OR 3.34, 95%CI [1.05, 10.62] for the groups with GRS=6, 7, and 8 or above, respectively (Figure 4). The trend of an increase in PCa risk according to the increase in GRS is very strong (p<0.0001, test for trend), and the risk for the highest GRS group (GRS>=8) compared with the lowest group (GRS<=1) was estimated to be increased more than 10-fold.

When the predictive performance of the GRS was evaluated using a receiver operating curve function, the area under the curve (AUC) obtained was 0.605 with a 95% CI ranged from 0.573 to 0.637 (Supplementary Figure 3).

## DISCUSSION

This is the first GWAS on PCa susceptibility in a Korean population. PCa is one of the most rapidly increasing cancers for Korean men over the past two decades. The incidence rate of PCa in Korea, however, is still lower than that of Caucasians or Africans, being 26.2 out of 100,000 person-year. Whether it is genes or the environment that contributes to this gap has been elusive. Our findings that all 5 lead genetic variants associated with PCa are replicated SNPs, support for genetic similarities, rather than differences. Interestingly, with only five markers the overall predictive value of the GRS was same as the PLCO study findings with 30 markers [20]. Emphasis on etiologic similarities of PCa over differences are equivalent with that for breast cancer [21]. Our findings suggest that it might be reasonable to expect that the epidemiology of PCa in Korea and probably in many East Asian populations would follow that of the populations with higher PCa incidence rates.

All 5 SNPs with exome-wide significance at the Stage I study were replicates of previous studies. Though the HumanExome BeadChip array targets the exome and rare variants, it inclusively contains GWAS tag SNPs and common variants grid where the detected SNPs fall into. All 5 susceptibility loci had been reported in European studies, and 3 loci were from other Asian studies. The effect size of the risk alleles was generally higher in this study than the previous findings. It is noteworthy that the larger effect size in the Korean population is statistically significant for two SNPs (rs1016343 and rs7837688) in the 8q24.21 region compared with other studies. These two SNPs in the 8q24.21 region are more common in Koreans or East Asians, whereas the other SNPs in GRS, showed lower frequencies in Koreans than those in Europeans or African ancestries. Variations in allele frequency across the population for these 5 susceptibility loci were relatively large, and the major/minor alleles were reversed for some of the SNPs. For example, for rs1512268 and rs7501939, the ancestral (major) alleles in African ancestry (both T allele) consist only 0.3 for Asians making these wild type as minor alleles. Considering the effect size and their allele frequencies of the susceptibility loci across the population, it is not likely that Korean men are genetically less susceptible to PCa. Our findings suggest that the current gap in the PCa occurrence across populations would be gradually reduced. The 8q24 region is currently considered the most important susceptibility region for PCa, accounting for about 8% of the two-fold increase in risk observed in first degree relatives [22]. The variants in this region spread across three haplotype blocks (Supplementary Figure 2) and probably contribute independently to PCa risk [22–25]. The 8q24 region is a gene “desert” with only a few predicted non-coding genes. The closest annotated protein coding gene, proto-oncogene MYC, is over 200 kb downstream from the nearest PCa risk variant. Multiple lines of evidence indicate that the 8q24 risk locus exhibits minimal RNA transcriptional output [26] and contains regulatory elements, especially enhancers [27].

Recently, functional roles of long non-coding RNAs (lncRNAs) have been suggested for PCa development; through epigenetic regulations/progression [28–30]. In the 8q24 region PRNCR1 is one of the highly expressed lncRNA genes reported in aggressive PCa cases. PRNCR1 is known to be associated with androgen receptor (AR)-related gene activation in prostate tumor tissues [31].

Currently, genetic data based prognostic biomarkers are available in PCa clinics notably Prolaris®, Decipher® and Oncotype Dx®, [32]. As of the year 2016, the established SNPs were estimated to explain 33% of the familial risk of PCa. [11]. Eeles et al showed that by using a polygenic risk score consisting of 68 SNPs associated with PCa men in the top 1% of the risk distribution have more than a four-fold increased risk for PCa compared with those with an average risk [33]. In our study, we recruited the Stage II case-control study to estimate the predictive value of the susceptibility loci. The highest GRS score group (GRS>=8) consisted of 2% (12 out of 586) of the cases and 0.7% of the controls (4 out of 548), and the increase in the PCa risk was 3.34 compared with those with average GRS scores. The predictive values of the risk loci from our study are largely compatible with other studies. A recent report suggested a possible application of this predictive ability to clinical practice. A polygenic risk score, including 35 established PCa risk SNPs, was recently shown to decrease the number of biopsies by 23% at a cost of 3% fewer cases detected in a Swedish cohort of men that underwent a biopsy of the prostate [34]. The discriminative power of the GRS in this study during the Stage II study was estimated to be 0.605. Even with the modest sample size, the risk size according to the GRS or the predictive value was compatible with other large studies, and our findings might potentially be applied for preventive measures.

Although this is the first report of a genomic association study of PCa in Korea, we admit some limitations. First, our study involved relatively modest number of cases which might have resulted in limited statistical power. We believe some loci that were previously replicated may have been missed out, and more importantly no new variants were found exceeding the exome-wide significance mainly because of this limited statistical power. In the same vein, all five GRS SNPs in this study used in order to predict prostate cancer risk were relatively common variants, not rare functional variants included in the Exome Array chip design. Second, the Stage II study analyzed selected SNPs to validate the predictive value of the Stage I findings, and thus, an official meta-analysis at a genome-wide scale was not possible. Third, the control groups were selected from a population-based cohort study, and the age of the controls was generally younger. It is unlikely, however, that the findings in this study were confounded by this age difference because genotypes are known to be randomly distributed, and the susceptibility loci in this study represent alleles that have significantly different distributions between populations and PCa patients.

In conclusion, we detected 5 risk variants for PCa in a Korean population, which were confirmed in many previously reported findings. Our findings suggest that similarities, rather than differences, in genetic susceptibilities might be a more important aspect across populations, but the impacts from the differences in the effect size, the allele frequency, and the LD block structure should be investigated further to elucidate the genetic cause of PCa. The addition of individualized calculated GRS effectively enhanced the accuracy of prediction. These results need to be validated in future studies.

## PATIENTS AND METHODS

### Ethics statement

This study was approved by our institutional review board (Seoul National University Bundang Hospital Institutional review board; IRB number, B-1312/232-302) and followed the rules stated in the Declaration of Helsinki. All participants provided written informed consent.

### Study design and populations

Our study was performed in a two-stage design as depicted in Figure 1. In Stage I, 1,001 PCa samples and 2,641 population controls were used to find the associated markers in the Korean population and examine the previously identified GWAS SNPs in the different ethnic populations. In Stage II, the GRS was calculated based on the validated SNPs from Stage I in order to determine its discriminative ability in an independent set of cases and controls.

For Stage I, we used the data from 988 PCa patients enrolled and treated in the Seoul National University Bundang Hospital from November 2003 to July 2013, and 2,641 controls from the Korean Association Resource (KARE) study as part of the Korean Genome and Epidemiology Study (KoGES). For all of the patients included in Stage I, blood specimens were prospectively collected throughout the course of the study, and serum PSA, clinical stages, biopsies Gleason scores, numbers of positive cores, and the pathological outcome data were recorded. Transrectal ultrasound-guided multi-core (≥12) biopsies were performed using an automatic firing mechanism. The collected prostate tissue samples were biopsied bilaterally near the base, mid-gland, and apex, with at least six biopsies per side. A total of 12 baseline biopsy cores were taken in all of the men, and additional biopsies of suspicious lesions were obtained if needed. Among the PC patients, 820 patients underwent RP in same hospital. All biopsy and RP specimens were analyzed by a single genitourinary pathologist. The controls were selected from 10,038 people total in the 40-69 years old residing in the cities of Ansung and Ansan recruited in 2001 through 2002. More detailed information about the study is available in a previously published article [35].

For the Stage II GRS evaluation, 516 cases and 548 controls were recruited from a tertiary institution, The Chungbuk University Hospital. The diagnosis of PCa was the same as described for the Stage I.

### Exome chip and quality control in stage I (GWAS)

The Stage I Exome-based discovery study was conducted using the HumanExome BeadChip 12v1-1 system (Illumina, Inc.; San Diego, CA), which includes 242,901 probes focused on protein-altering variants (non-synonymous, stop and splice) selected from exome and whole-genome sequences [36, 37]. Details about SNP content and selection strategies can be found at the exome array design webpage (http://genome.sph.umich.edu/wiki/Exome_Chip_Design). Genotype calling was performed using Illumina's GenTrain version 2.0 clustering algorithm with the GenomeStudio software (V2011.1). Cluster boundaries were determined using Illumina's standard cluster file. To improve the accuracy of variant calling, manual reclustering and visual inspection were conducted for genotypes based on the CHARGE clustering method [36]. Sample quality control was carried out to exclude samples with genotyping rates < 95%, heterozygosity, and cryptic relatedness. Markers were excluded based on the following criteria: 1) monomorphic in our samples, 2) missing call rate > 5%, 3) significant deviation from the Hardy-Weinberg equilibrium (*P* < 1.0 × 10^-6^). The 988 case and 2,641 control subjects and 60,276 variants remained after quality control were taken forward for subsequent analysis.

### Genotyping for *de novo* replication

To have more numbers of loci available to choose from when selecting sets of informative markers for the GRS construction with exome-wide significant lead markers, at first, 5 exome-wide significant lead SNPs (*P* < 1.0 × 10^-4^) from the Stage I were selected to genotype in the Chungbuk samples for the replication. The genotyping of these SNPs was performed using the Fluidigm 192.24 Dynamic Array TM IFC and Biomark HD systems. Duplicates and negative controls were included in each 96-well plate for quality control. Technicians blinded to the sample status performed the genotyping. The average concordance rate between the duplicate samples was >99%.

### GRS construction and risk assessment

To construct a GRS using PCa susceptibility loci, we considered the five exome-wide significant lead SNPs (*P* < 8.30 × 10^-7^) from each LD block which were also previously validated within the other ethnic groups. The cumulative number of risk alleles was calculated using an additive model (0 for homozygous of non-risk alleles, 1 for heterozygous of alleles and 2 for homozygous of the risk alleles for each SNP). The GRS was calculated by a non-weighted sum of the risk allele counts (non-weighted GRS), because the model with non-weighed GRS showed better model fitting than that with odds raio-weighted GRS in the initial explanatory model. Then, we obtained estimates of the area under the receiver-operating characteristic curve (AUC) to evaluate its discriminative ability in an independent set of cases and controls from the Chungbuk University Hospital.

### Statistical analysis

Single-variant association tests were analyzed using EPACTS v3.2.4 (http://genome.sph.umich.edu/wiki/EPACTS), PLINK (http://zzz.bwh.harvard.edu/plink/) and SAS programs (version 9.1; SAS institute Inc., Cary, NC, USA). The data were analyzed using an unconditional logistic regression to calculate an odds ratio (OR) as an estimate of the relative risk of PCa susceptibility with the SNP genotypes. For multiple comparisons, a statistically significant threshold (*P* < 8.30 × 10^-7^) was used based on the Bonferroni correction.

## SUPPLEMENTARY MATERIALS FIGURES AND TABLE




