# Supplementary material for: Exome-based genome-wide association study and risk assessment using genetic risk score to prostate cancer in the Korean population
Source: Oncotarget. 2017 Mar 24;8(27):43934–43. doi: 10.18632/oncotarget.16540 (PMC5546451; doi:10.18632/oncotarget.16540)
Supplement: Supplementary file 1 [file oncotarget-08-43934-s001.pdf]

Exome-based genome-wide association study and risk assessment using genetic risk score to prostate cancer in the Korean population

SUPPLEMENTARY FIGURES AND TABLE

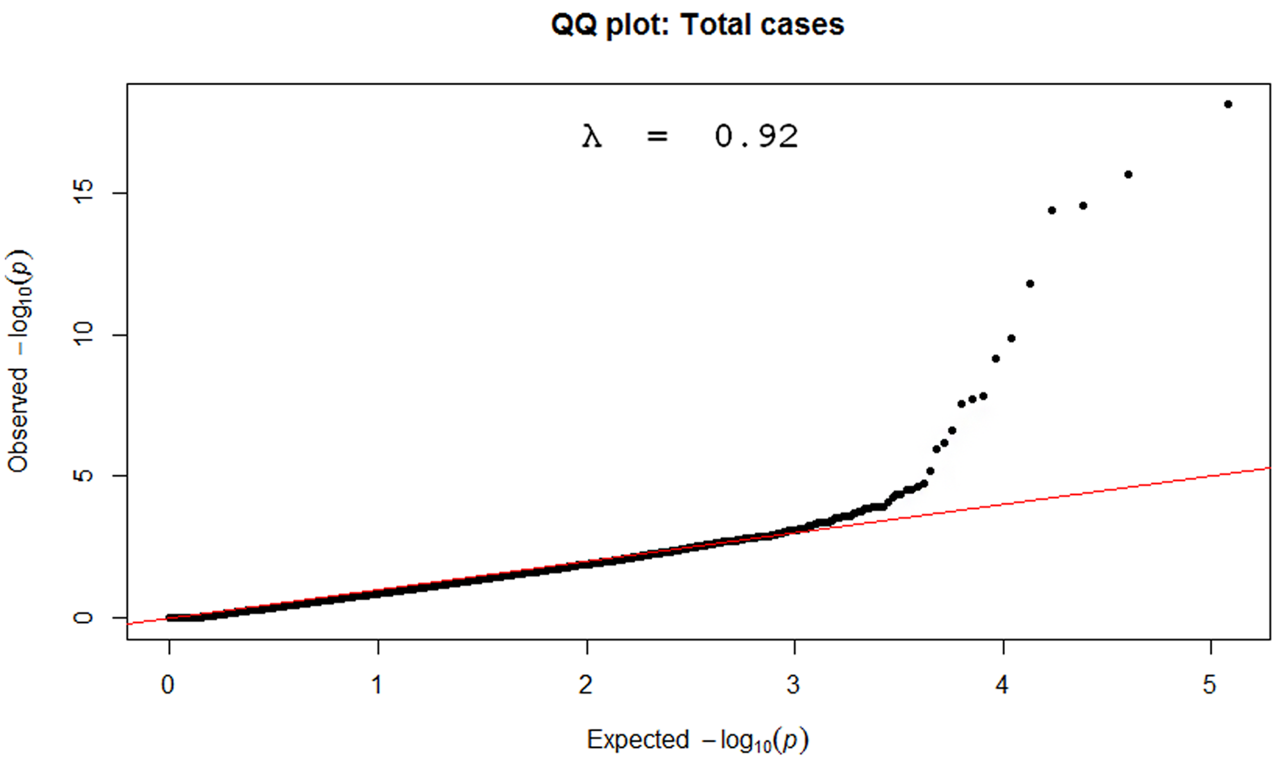

Supplementary Figure 1: Quantile-Quantile (QQ) plot. The observed p-values (y-axis) were compared with the expected p-values (x-axis) under the null distribution on the scale of –log10. The overall inflation factor (lambda) was estimated to be 0.92 showing little evidence of possible Type I errors.

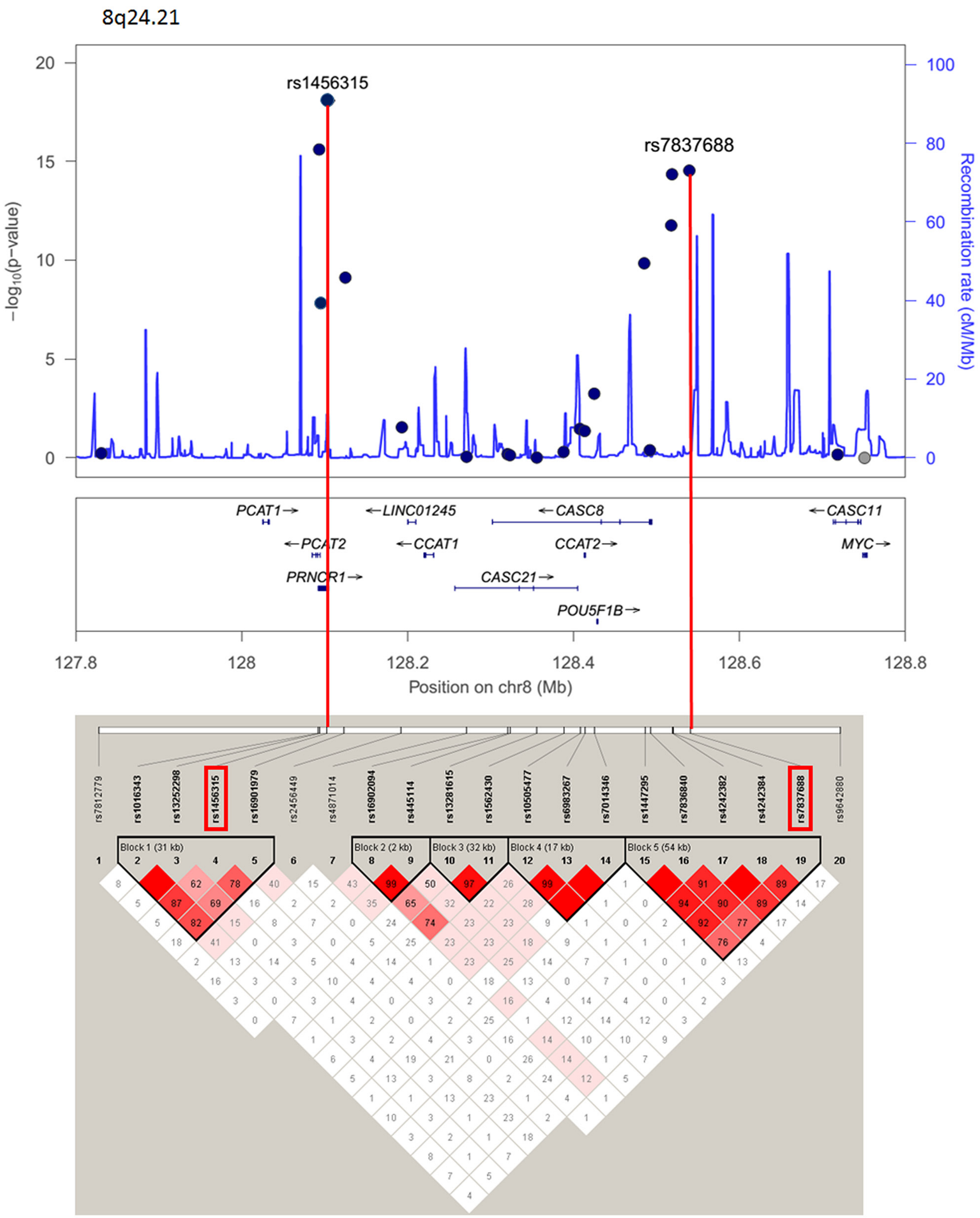

Supplementary Figure 2: Regional plot paired with a linkage disequilibrium (LD) block of the chromosomal region 8q24.21. Two of the top SNPs (rs1456315 and rs7837688) associated with prostate cancer are located in the 8q24.21 region.

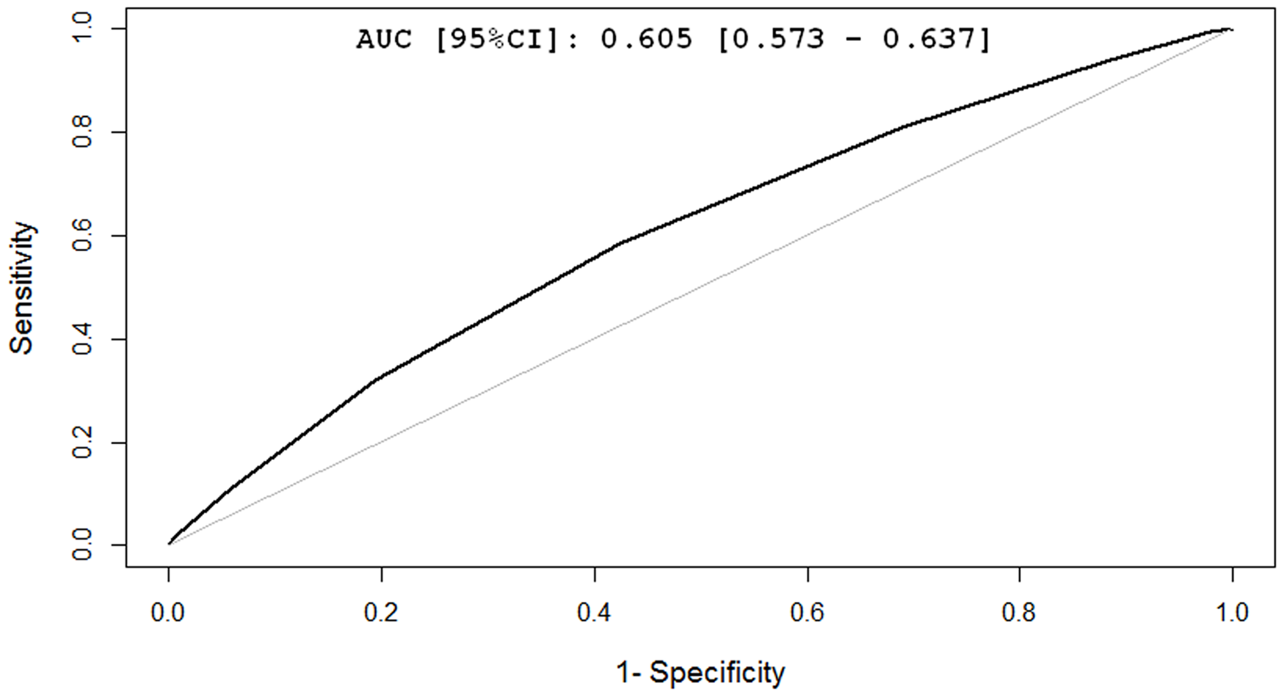

Supplementary Figure 3: Receiver operating characteristic (ROC) curve showing the discrimination between the cases and controls by the genetic risk scores. Area under the curve (AUC) was 0.605 for the ROC.

Supplementary Table 1: Selected target 22 SNPs associated with prostate cancer in discovery GWAS stage ($p < 1 \times 10^{-4}$).

(See Supplementary File 1)
