# Supplementary material for: Exome-based genome-wide association study and risk assessment using genetic risk score to prostate cancer in the Korean population
Source: Oncotarget. 2017 Mar 24;8(27):43934–43. doi: 10.18632/oncotarget.16540 (PMC5546451; doi:10.18632/oncotarget.16540)
Supplement: Supplementary file 2 [file oncotarget-08-43934-s002.doc]

Supplementary Table 1. Selected target 22 SNPs associated with prostate cancer in discovery GWAS stage (*p* < 1 × 10^-4^)

| SNPID | Chr | location | Alleles | Gene | MAF | p-value |
| --- | --- | --- | --- | --- | --- | --- |
| rs1016343 | 8q24.21 | intron | A/G | PRNCR1 | 0.3341 | 1.29E-16 |
| rs13252298 | 8q24.21 | intron | G/A | PRNCR1 | 0.2761 | 1.28E-08 |
| rs1456315 | 8q24.21 | intron | G/A | PRNCR1 | 0.2647 | 3.11E-19 |
| rs16901979 | 8q24.21 | intergenic | A/C | PRNCR1 – CASC19 | 0.2468 | 5.91E-10 |
| rs1447295 | 8q24.21 | intron | A/C | CASC8 | 0.1780 | 9.99E-11 |
| rs7837688 | 8q24.21 | intergenic | A/C | CASC8 - CASC11 | 0.1572 | 1.23E-15 |
| rs4242382 | 8q24.21 | intergenic | A/G | CASC8 - CASC11 | 0.1912 | 1.05E-12 |
| rs4242384 | 8q24.21 | intergenic | C/A | CASC8 - CASC11 | 0.1799 | 2.05E-15 |
| rs1512268 | 8p21.2 | intergenic | A/G | NKX3-1 | 0.3253 | 1.66E-08 |
| rs339331 | 6q22.1 | intron | G/A | RFX6 | 0.3461 | 6.21E-06 |
| rs4430796 | 17q12 | intron | G/A | HNF1B | 0.2983 | 1.03E-06 |
| rs7501939 | 17q12 | intron | A/G | HNF1B | 0.2684 | 2.40E-08 |
| rs2735839 | 19q13.33 | intron | A/G | KLK3 | 0.3699 | 5.86E-07 |
| rs75647314 | 4q13.2 | intron | A/C | TMPRSS11B | 0.0074 | 1.67E-29 |
| rs1048167 | 5q13.3 | nonsynonymous | A/G | GFM2 | 0.0047 | 1.29E-18 |
| rs11147922 | 13q14.11 | intron | G/A | ENOX1 | 0.3603 | 2.75E-05 |
| rs28484999 | 16q23.2 | intron | A/G | CDYL2 | 0.0209 | 5.96E-26 |
| rs6901250 | 6q22.1 | synonymous | G/A | GPRC6A | 0.4971 | 5.68E-05 |
| rs636252 | 6q22.1 | intergenic | G/A | GPRC6A – RFX6 | 0.4825 | 2.80E-05 |
| rs2016588 | 6q25.3 | intergenic | A/G | RSPH3 - TAGAP | 0.4938 | 1.75E-05 |
| rs57006764 | 12p13.31 | nonsynonymous | A/G | PZP | 0.4282 | 3.95E-05 |
| rs149008055 | 15q26.1 | synonymous | G/A | FANCI | 0.0063 | 3.19E-05 |

OR = odds ratio; SNP = single nucleotide polymorphism
